# Newly identified risk factors for MRSA carriage in The Netherlands

**DOI:** 10.1371/journal.pone.0188502

**Published:** 2017-11-30

**Authors:** W. S. N. Lekkerkerk, A. Haenen, M. A. B. van der Sande, T. Leenstra, S. de Greeff, A. Timen, A. Tjon-a-Tsien, J. H. Richardus, N. van de Sande-Bruinsma, M. C. Vos

**Affiliations:** 1 Department of Medical Microbiology and Infectious Diseases, Erasmus MC, University Medical Center Rotterdam, Rotterdam, The Netherlands; 2 RIVM, National Centre for Health and Environment, Bilthoven, The Netherlands; 3 UMCU, Julius Centre, Utrecht, The Netherlands; 4 The Institute of Tropical Medicine, Antwerp, Belgium; 5 Municipal Public Health Service Rotterdam Rijnmond, Rotterdam, The Netherlands; 6 Department of Public Health, Erasmus MC, University Medical Center Rotterdam, Rotterdam, the Netherlands; Universitatsklinikum Hamburg-Eppendorf, GERMANY

## Abstract

**Objectives:**

To elucidate new risk factors for MRSA carriers without known risk factors (MRSA of unknown origin; MUO). These MUO carriers are neither pre-emptively screened nor isolated as normally dictated by the Dutch Search & Destroy policy, thus resulting in policy failure.

**Methods:**

We performed a prospective case control study to determine risk factors for MUO acquisition/carriage (Dutch Trial Register: NTR2041).

Cases were MUO carriers reported by participating medical microbiological laboratories to the RIVM from September 1^st^ 2011 until September 1^st^ 2013. Controls were randomly selected from the community during this period.

**Results:**

Significant risk factors for MUO in logistic multivariate analysis were antibiotic use in the last twelve months, aOR 8.1 (5.6–11.7), screened as contact in a contact tracing but not detected as a MRSA carrier at that time, aOR 4.3 (2.1–8.8), having at least one foreign parent, aOR 2.4 (1.4–3.9) and receiving ambulatory care, aOR 2.3 (1.4–3.7). Our found risk factors explained 83% of the MUO carriage.

**Conclusions:**

Identifying new risk factors for MRSA carriers remains crucial for countries that apply a targeted screening approach as a Search and Destroy policy or as vertical infection prevention measure.

## Introduction

In The Netherlands MRSA prevalence is low, measured at 0.12% at hospital admission in 2005–2007[1] and 0.8% in Dutch outpatients in the Dutch-German border region in 2012.[2] Among *S*.*aureus* blood-cultures the MRSA prevalence was 1.0% (24/2,386).[3] To keep MRSA prevalence low, prudent use of antibiotics is instigated and a Search and Destroy policy (S&D) is in place. S&D consists of screening of defined risk patients (Table 1) at hospital admission, and by pre-emptive isolation of them pending the screening results.[4] Colonized patients and healthcare workers are treated with strict treatment regimens to eradicate the carriage of MRSA. [4]

**Table 1:** Risk categories in the Dutch WIP guideline on MRSA.

January 2007 (updated: March 2008)				December 2012			
Patients		Healthcare workers		Patients		Healthcare workers	
**Category 1**		**Category 1**		**Category 1**		**Category 1**	
-	Proven carrier status of MRSA	-	Proven carrier status of MRSA	-	Proven carrier status of MRSA	-	Proven carrier status of MRSA
				-	Follow-up after MRSA eradication therapy (3 follow-up culture-sets)	-	Follow-up after MRSA eradication therapy (3 follow-up culture-sets)
						-	Undergoes MRSA eradication treatment
**Category 2**		**Category 2**		**Category 2**		**Category 2**	
-	Nursed < 2 months > 24h in a foreign hospital	-	Unprotected contact with MRSA carrier	-	< 2 months ago unprotected contact with MRSA carrier inside (as part of contact tracing) or outside (household members, partners, caretakers of MRSA carriers) the hospital	-	Nursed < 2 months > 24h in a foreign care facility
-	Nursed < 2 months < 24h in a foreign hospital with the following risk factors at arrival in a Dutch hospital: operation, infection, catheter or drains present	-	Hospitalized < 2 months ago in a foreign hospital, were operated abroad, received a drain or catheter, were intubated, have skin lesions or possible infectious sources such as abscesses or furuncles.			-	Nursed < 2 months < 24h in a foreign care facility with at least one of the following risk factors: operation abroad, chronic infection or persistent skin lesions, presence of abscesses or furuncles at hospitalization in the Netherlands.
-	Patient from department (hospital or nursing home) with an ongoing MRSA outbreak			-	Nursed < 2 months < 24h in a foreign care facility with at least one of the following risk factors: operation abroad, chronic infection or persistent skin lesions, presence of abscesses or furuncles at hospitalization in the NL.		
-	Adopted children regularly hospitalized or visiting the hospital						
-	Foreign patient at dialysis unit			-	Foreign dialysis patients		
-	Share a room with unexpected MRSA carrier			-	Stayed < 2months ago in a Dutch care facility (unspecified) with an ongoing MRSA outbreak on the department		
-	After MRSA eradication therapy but before follow-up culture-sets are taken			-	Adopted children from abroad living in the Netherlands		
-	Contact with live pigs or veal calves			-	Contact with industrial, live pigs, veal calves or broiler chickens regardless whether this contact was professional or not, and/or lives on such a farm.		
**Category 3**		**Category 3**		**Category 3**		**Category 3**	
-	Dutch dialysis patients dialyzed abroad	-	Protected contact with MRSA carriers	-	Unprotected contact with MRSA positive HCW < 2 months ago	-	Persistent exposure with a negative MRSA test less than three months ago.
-	First year after MRSA eradication therapy with MRSA negative follow-up culture-sets	-	< 2 months ago worked abroad >24h in a hospital or nursing home	-	Dutch dialysis patients dialyzed abroad < 2 months ago	-	Unprotected contact with MRSA positive patient < 2 months ago inside or outside the hospital
-	Nursed > 2 months in foreign hospital with persistent skin infections or risk factors	-	First year after MRSA eradication therapy with MRSA negative follow-up culture-sets	-	Nursed > 2 months in foreign hospital with persistent skin infections or risk factors	-	< 2 months ago > 24h patient-related activities in a foreign care facility
				-	First year of follow-up after MRSA eradication therapy and the first three negative follow-up culture-sets	-	Guided patients < 2 months ago from a foreign to a Dutch care facility without isolation precautions
				-	Persistent exposure with a negative MRSA test less than three months ago.	-	Carrier with uncomplicated MRSA who was negative before the start of MRSA eradication therapy
**Category 4**		**Category 4**		**Category 4**		**Category 4**	
-	Nursed > 2 months ago in a foreign hospital without persistent skin lesions or risk factors	-	Successful MRSA eradication therapy > 1 year ago. Follow-up culture-sets remained MRSA negative	-	None of the above categories applies	-	None of the above categories applies
-	Stayed < 24h in a foreign hospital without risk factors or operations	-	Negative follow-up culture-set after protected contact with MRSA carrier				
-	On a department with MRSA where adequate precautions were taken						
-	Negative follow-up culture-sets a year after MRSA eradication therapy ended						

HCW: Healthcare worker. For the trawling and case control questionnaires, the risk categories of the 2007–2008 WIP guideline were used. In 2015, the RIVM added as risk factor a refugee visiting a Dutch hospital who had been in a refugee camp less than two months before (category 2/3).

One of the revisions on MRSA risk groups, was due to the discovery of livestock-associated MRSA with sequence type 398 (LA-MRSA ST398) in The Netherlands.[5] The risk groups for S&D were subsequently updated with pig and veal calf farmers. In 2016, LA-MRSA accounted for 26% (892/3,478) of MRSA isolates.[3]

Apart from the discovery of LA-MRSA, it appeared that the proportion of reported MRSA without known risk factors, thus not defined as risk patients, became substantial. In 2008–2009, 25% (1350/5545) of all MRSA were reported as MRSA without known risk factors [6] In 2016, this has increased to 38% (810/2,121). [3] The MRSA without known risk factors were named MRSA of Unknown Origin or MUO. MUO are per definition unexpected and are mostly detected in clinical samples. However, in screening samples on MRSA, MUO can be detected as well. This is the case when the found MRSA genotype does not match the MRSA genotype of the index person.[6]

We started a nation-wide study to explore the risks and causes of MUO, so the defined risk groups in S&D policy can be updated and unnoticed dissemination of MRSA in healthcare settings and the community can be stopped. In this paper, we report the results from our prospective case control study to determine the risk factors for carriage of MUO.

## Methods

### MRSA surveillance

In the Netherlands, all MRSA are detected either through active surveillance screening or in a clinical sample and are mandatorily sent to the Dutch National Institute for Public Health and the Environment (RIVM). Yearly, around 3,000 isolates are submitted to the RIVM.[7] Along with the isolate, risk factors for MRSA carriage, as defined in the MRSA guideline by the Dutch Working party on Infection Prevention (WIP), are reported to the RIVM by standard questionnaire.[8, 9] Any person detected with MRSA and reported with one or more risk factors as described in this WIP guideline is defined as MRSA of Known Origin (MKO). Any person detected with MRSA lacking these risk factors, is defined as MRSA of Unknown Origin (MUO).

### Trawling study

A trawling questionnaire was forwarded by Dutch Medical Microbiological Laboratories (MML) in 2010 to all MUO carriers reported to the RIVM in 2009. The retrospective trawling questionnaire was set-up to learn which risk factors could be involved with MUO to narrow and specify the number and kind of questions in the case control questionnaire, as well to choose the best control group for the case control study. To prevent recall bias, the maximum timespan for events in the past that had to be recalled by trawling study participants was limited to two years.

To confirm that these MUO carriers were not misclassified MKO carriers, the questionnaire included questions on the described risk factors for MRSA in the Dutch WIP guideline on MRSA (Table 1). Furthermore, the questionnaire included questions on occupations, sports, leisure, social habits and lifestyles, and risk factors in other populations described in the literature. (PubMed at 01-01-2010, search keywords ‘MRSA’ and ‘risk factor’).

Excluded cases were non-responders, the deceased, potential cases that lacked an address or were misclassified as MKO for various reasons. Included cases were all MUO carriers not misclassified as MKO upon return of the questionnaire.

Questionnaire data was analysed as described under ‘statistics’, and the results were interpreted to update the questionnaire and define the best controls.

### Case control study

#### Study population

The study population consisted of patients detected with MRSA but without known risk factors (MUO) and population controls. The sample site and frequency of detection was not taken into account. In the Netherlands, persons detected with MRSA are all included in the national MRSA database, regardless of sample site, infection or indication for sampling.

To determine the risk factors for MUO, we approached cases and controls with questionnaires (S1 Text). Case control study participants who answered ≥95% questions of the total of 43 questions and of whom informed consent was obtained, were rewarded with 25 euros and enrolled as case or control.

To detect an odds ratio (OR) of 2 or higher with 80% power and an alpha of 0.05, we aimed to enrol 500 cases and 1,000 controls (1:2 case-control ratio), based on an estimated 700 MUO reported to the MRSA surveillance in two years (on a total of ±3,000 MRSA carriers reported per year).

#### Case definition

Potential cases were MRSA carriers, reported by the participating MML (medical microbiologist or infection prevention personnel) as MUO to the RIVM for the MRSA surveillance from September 1^st^, 2011 until September 1^st^, 2013. Before sending the questionnaire, assumed MUO cases were checked on the following exclusion criteria: death, lack of address or misclassification (of a MKO as a MUO). Upon return of the questionnaire, we checked once more for misclassification. True MUO were included as case.

#### Control definition

Selecting the right controls was based on the results from the trawling study (see results below). The best control group was considered to be unmatched community controls. This choice was based on the fact that the MUO were selected from the RIVM database, which contained not only MRSA carriers detected at hospitals, but also those detected by general practitioners or in long-term care facilities. Furthermore, MUO carriers were shown to be a diverse group of carriers.

The controls were randomly chosen from 60 Dutch municipalities from all over of The Netherlands. These 60 municipalities were a national representative subset of all 415 municipalities in The Netherlands, and contained large, middle and small municipalities.

#### Inclusion procedure

Every two weeks a printout was made in Microsoft Excel of the newly reported MUO to the RIVM. Questionnaires for MUO carriers were sent to participating MML (S1 Text). The MML checked the carriers on exclusion criteria before forwarding the questionnaires to the MUO carriers.

After three weeks, non-responders were once again approached by questionnaire or by telephone. Returned questionnaires of both cases and controls were checked to exclude any MKO carriers misclassified as MUO. Misclassifications were not included as cases.

Controls were sent a questionnaire by mail. After three weeks non-responders were approached once more by mail or if possible by telephone.

This study was approved by the medical ethical committee at the Erasmus MC and registered in the Dutch Trial Register under NTR2041. Written informed consent was requested from both cases as controls, before participating in this study by questionnaire. Data were aggregated and anonymized before analysis.

#### Statistics

Questionnaire data were analysed with SAS Enterprise Guide (version 4.2 by SAS Institute Inc., North Carolina, USA), using descriptive, univariate (2x2 tables and Fisher’s Exact test) and multivariate analysis (multiple regression logit model, backward elimination with significance level of 0.05 to stay in the model, with and without dummy variables).

## Results

### Trawling study

**Fig 1:**
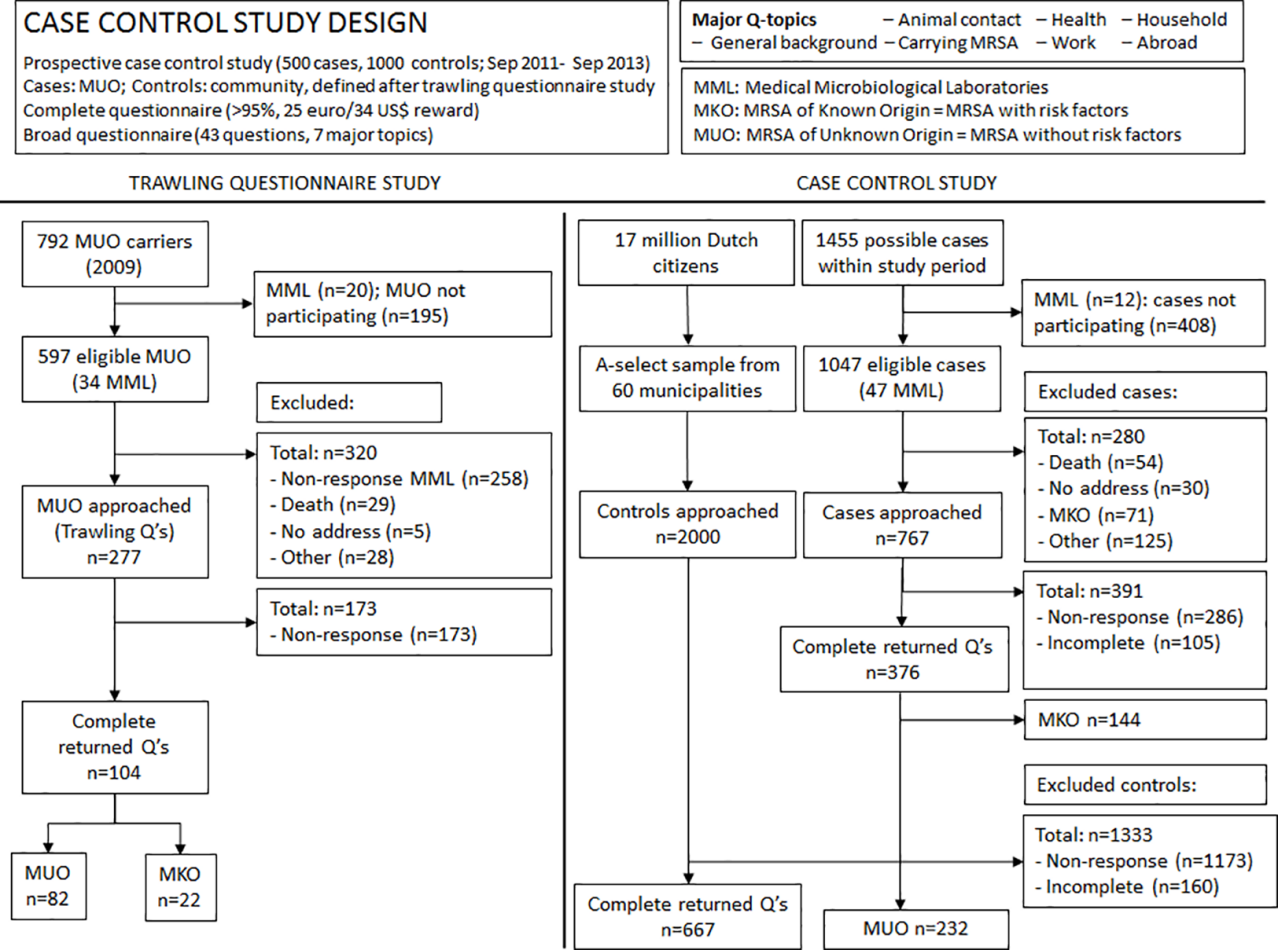
Flowcharts for trawling and case control studies.

Of the reported 794 MUO to the MRSA surveillance, only 277 fulfilled inclusion criteria and could be approached by questionnaire through participating MML (Fig 1). Of these 37% (104/277) responded and all age groups were present. Of the 104 returned questionnaires, 22 were MKO, and thus misclassified as MUO and the remaining 82 were MUO.

Fifty-two percent (43/82) of MUO carriers were male and none of the MUO were healthcare workers. Sixty-six percent (54/82) of MUO were patients detected in the hospital. However the other MUO carriers were detected by general practitioners or community-based healthcare institutions other than hospitals.

### Case control study

Between September 1^st^ 2011 and September 1^st^ 2013 1,455 MUO were reported to the RIVM and 767 MUO were approached by questionnaire (Fig 1). The response rate among cases was 49% (376/767), that of controls 33% (667/2,000). Of the 376 returned questionnaires, 38% (144/376) of cases turned out to be MKO, thus misclassified as MUO, leaving 232 cases for analyses. None of these 232 cases were healthcare workers.

Comparing 232 cases with 667 controls, some risk factors, such as hospitalization of a household member, chronic disease, and carriage of ST398 MRSA without professional contact with pigs/veal calves or other farm animals, were significant in univariate analysis, but not in the final regression model (Table 2).

**Table 2:** Multivariate logistic regression for MUO risk factors.

Risk factor	MUO (n = 232)	Controls (n = 667)	p-value	aOR^a^	95%CI
ST398 but without professional contact with pigs/veal calves or other farm animals*	18	0	n.s.	1.8	(0.6–5.2)
Hospitalization within household^b^	84	157	n.s.	1.1	(0.7–1.7)
Chronic disease	85	135	n.s.	1.4	(0.9–2.0)
Antibiotic use in last 12 months	150	139	< 0.01	8.1	(5.6–11.7)
Screened as part of a contact tracing but not found to be a MRSA carrier at the time	24	23	< 0.01	4.3	(2.1–8.8)
At least one foreign parent	48	71	< 0.01	2.4	(1.4–3.9)
Ambulatory care received	55	66	< 0.01	2.2	(1.4–3.7)

aOR: adjusted Odds Ratios; CI: Confidence Interval

* before 2012 only professional contact to pigs/veal calves was a risk factor. After 2012 any contact to pigs, veal calves and broiler chickens became a risk factor. But presence on a farm is not a risk factor per se, unless at the farm they have pigs, veal calves or broiler chickens.

^a^Logistic regression model with backward elimination containing the following factors: no professional contact with pigs/veal calves or other farm animals, antibiotic use in the preceding 12 months, chronic disease, not detected with contact tracing, at least one foreign parent, hospitalization within the household and ambulatory care. The R2max of the model was 0.29, while the AUC was 0.79.

^b^These factors were univariate significant, as well as possible confounders for receiving ambulatory care.

Significant factors in the multivariate logistic regression model were antibiotic use in the last twelve months aOR 8.1 (95%CI 5.6–11.7), screened as contact in a contact tracing but not detected as a MRSA carrier at the time aOR 4.3 (95%CI 2.1–8.8), having at least one foreign parent aOR 2.4 (95%CI 1.4–3.9), and receiving ambulatory care aOR 2.3 (95%CI 1.4–3.7).

The most frequently used antibiotics by cases and controls were ß-Lactam-antibiotics. There was no significant difference (p = 0.9) between cases and controls for ß-Lactam-antibiotics use in general, although there was a significant (OR 5.7; 95%CI 1.4–23.1) difference for the use of Amoxicillin/clavulanic acid (13.2% among cases (7/53) versus 2.6% (3/116) among controls).

Among ambulatory care use, home care was the most common, and MUO carriers were significantly more exposed (OR 3.3; 95%CI 1.5–6.9) to it.

Eighty-three percent of all MUO could be explained by the found independent risk factors in multiple regression model. (Table 3).

**Table 3:** Risk factors for MUO.

Risk factors			MUO (%; n = 232)	
	All cases with the risk factor:			
		Antibiotic use in the last 12 months	150	(64.7)
		Screened as part of a contact tracing but not found to be a MRSA carrier at the time	24	(10.3)
		At least one foreign parent	48	(20.7)
		Ambulatory care received	55	(23.7)
	Number of cases that only have this one risk factor			
		Antibiotic use in the last 12 months	52	(22.4)
		Screened as part of a contact tracing but not found to be a MRSA carrier at the time	5	(2.2)
		At least one foreign parent	8	(3.4)
		Ambulatory care received	6	(2.6)
	Number of cases that only have one risk factor		71	(30.6)
	Number of cases with a combination of 2 or more of the above risk factors		121	(52.2)
	Total cases of MUO explained by these risk factors		192	(82.8)
	Remaining unexplained MUO		40	(17.2)

MUO carriers had a single risk factor in 30.6% (71/232) and had in 52.2% multiple risk factors. Among those MUO with a single significant risk factor, antibiotic use in last twelve months accounted for 22.4%, at least one foreign parent for 3.4%, ambulatory care received for 2.6%, and screened as part of a contact tracing but not found to be a MRSA carrier at the time for 2.2%.

## Discussion

We identified the following independent risk factors for MUO: antibiotic use in the preceding 12 months, receiving ambulatory care, and being screened for MRSA in contact tracing but not having been detected at the time. Travelling abroad was not a risk factor, although we found a significant association with having a foreign parent.

In literature, antibiotic use in the last twelve months has been described before as risk factor for the general population; as a risk factor for MRSA carriage in children [10, 11], and within households where carriers were present [12]. Also, a systematic review showed a association between antibiotic exposure to quinolones, glycopeptides, cefalosporins and beta-lactams and an increased risk of MRSA isolation in adults.[13, 14] These former findings were confirmed by our study, as we found a significant difference in amoxicillin/clavulanic acid use between cases and controls. Greater use of amoxicillin/clavulanic acid may be due to more infections among MUO carriers compared to the controls. We cannot rule out this possibility as we did not measure the number of infections among MUO, since submission of an infection isolate to the MRSA database is preferred but not obliged. The Netherlands has the lowest use of all antibiotics in the European Union. In 2013, 2015 and 2017, it was 10.8, 10.7 and 10.4 defined daily doses/1000 inhabitants/day respectively.[3, 15]

Interestingly, having been screened as part of a contact tracing in the past, but not detected at that time, was a significant risk factor for MUO carriage. This is an important risk factor for countries with S&D policy, as this policy aims to identify all people at risk, including contacts. Explanations for this risk factor could be a too low sensitivity of MRSA culture, missing sampling sites, or when sampling occurs too early after exposure, and is not follow-upped with repeated sampling. For this reason it is also recommended to sample healthcare workers on start of their next duty instead of immediately after unprotected contact with a MRSA carrier. The current guideline indicates one set of samples from nose, throat, rectum and wounds when present. The guidelines assumes a sampling frequency of one set to be sufficient. There are no indications in the guideline on the timing of sampling of contacts after exposure when tracing MRSA contacts. Indeed, in our previous study we showed that carriage is not always detectable in each sample moment when sampling after MRSA eradication therapy to monitor MRSA recurrence.[16] Further studies on reliability of contact tracing should be conducted, especially in regards to the number and sample sites of cultures when screening for contacts.

Having at least one foreign parent, was also one of the significant risk factors. Possibly, an immigration background from countries with a higher MRSA prevalence may result in a higher exposure to MRSA by visiting or close contact within the family. Especially those countries with higher levels of CA-MRSA. This is in line with findings from Denmark which showed 40% of affected individuals CA-MRSA infections with certain CA-MRSA clones had a positive family history related to foreign regions where such clones were predominant. [17] Furthermore, the MRSA prevalence among actively screened asylum seekers (refugees/immigrants) in The Netherlands was 9.7% (87/898). [18] Similar to findings in Germany, but much higher than the prevalence in the general population at hospital admission.[18, 19] In 2015, the RIVM added as risk factor a refugee visiting a Dutch hospital who had been in a refugee camp less than two months before (category 2/3).[20] It is possible that a limited understanding of the Dutch language prevented refugees from participating. If this is the case then our aOR for having at least one foreign parent, is an underestimation. Our national MRSA database currently contains *spa*, MLVA and PVL data. But this typing data, along with scarce epidemiological data, is currently not sufficient to link MUO to outbreaks abroad or transmission or sources occurring outside a Dutch health care centre. Furthermore, in this study we did not analyse the typing data, including PVL. As we did so in a previous study, and learned that PVL positive MRSA isolates were significantly larger among MUO than among MKO.[6]

Ambulant or home care exposure are scarcely published in literature as risk factor for MRSA carriage, as only ambulatory care facilities in Germany were described and designated as a reservoir for dissemination.[21] Theoretically, transmission of MRSA through ambulatory care could be possible, thus creating new MUO carriers. This finding necessitates further investigation in the future in The Netherlands, as ambulatory care facilities are becoming more important in a population with a growing segment of the elderly.

The use of arbitrary cut-offs in risk factor definitions could theoretically result in MUO. However, after multivariate analysis we found no significant risk factors related to arbitrary cut-offs in risk factor definitions, such as ‘less than two months ago’ in case of a visit to a hospital abroad.

We could not confirm poultry consumption or scuba gear sharing as risk factors as found in the study by van Reijen et al.[22] Possible explanations for the difference could be due to design, different selection and inclusion criteria of cases and controls, difference in questioning, and the number and selection of participating MML. Continuous analyses of MUO and its risk factors in the future will be necessary, not only to measure the effect of new policy implementation, but also to elucidate differences in outcome between studies.

Two risk factors published in recent years, fine air particles for Cystic Fibrosis (CF) patients and livestock-density for livestock-associated MRSA, were not included in our case control questionnaire.[23, 24] However, we think the impact of the absence of these risk factors in the questionnaire is minimal.

The use of standardized questionnaires, representative nationwide participation and community controls were study strengths which allows us to generalize results for all MUO in The Netherlands. Due to low national MRSA prevalence at hospital entry,[1] the odds of including MRSA carriers among the controls were very low.

The confines of the MRSA surveillance database, the lack of exact data on infection/carriage, the necessity to contact and possible recall bias were limitations of our study.

We aimed to enrol 500 cases and 1,000 controls based on an estimation of 700 MUO per two years to detect odds ratios of two or higher with 80% power. Inclusion of MUO was more difficult due to misclassification of some MUO and a lower willingness to participate in the study than expected. We therefore ended up including 232 cases and 667 controls, which was still enough to detect OR of 2 or higher with an 80% power and an alpha of 0.05, due to a larger number of controls per case.

In the trawling questionnaire study, as well as in the case control study, we found there was misclassification of MKO as MUO, inflating the number of MUO in the MRSA surveillance. In the future, more effort is needed to detect the presence of risk factor before classifying a carrier as MUO, and thus registration of MKO or MUO in the RIVM database should be improved to reduce the number of misclassified MUO. Currently, 38% of total reported MRSA are reported as MUO[3, 7], underlining its significance. Even after correction for misclassification, MUO is estimated to be a fourth of total MRSA reported to the surveillance each year.

Some of the newly defined risk factors, such as antibiotic use, can be common (Table 2) and would have low specificity when included into S&D risk groups. Other risk factors, such as being part of contact tracing in the past, could result in changes to the national guideline in regards to sampling frequency and timing. To determine the probability of MRSA carriage more precisely in the future, the known risk factors (current ones in the WIP guideline and from this study) should be analysed by creating risk tables or an algorithm. The presence of a single or combined risk factors could thus lead to targeted action such as screening or screening in combination with isolation on admission. Such a probability analysis could be subject of a next study.

For countries that apply a S&D approach as vertical infection prevention approach, MUO identification and elucidation is important. In a targeted screening approach as in S&D, persons at risk for MRSA carriage are identified (targeted) by means of risk factors. Monitoring MRSA is necessary to evaluate the effect of policy adjustments and any epidemiological changes that may give rise to new risk factors. Antibiotic use in the preceding 12 months, receiving ambulatory care and having at least one foreign parent, are common risk factors with limited practicality, but could still prove useful when combined to determine the probability of MRSA carriage risk based on a risk table and algorithm.

In conclusion, risk factors for MUO were mainly healthcare related despite MUO carriers not always being hospital-associated. Our new risk factors elucidated 83% of MUO, bringing us a step closer to preventing MUO from undermining successful S&D policy.[25]

## Supporting information

S1 Text Case control questionnaire. (DOCX)

S2 Text SAS log–New risk factors MRSA. (TXT)

S3 Text SAS report–New risk factors MRSA. (PDF)

S1 Table Underlying data–New risk factors MRSA. (XLSX)
