# Supplementary material for: Newly identified risk factors for MRSA carriage in The Netherlands
Source: PLoS One. 2017 Nov 30;12(11):e0188502. doi: 10.1371/journal.pone.0188502 (PMC5708665; doi:10.1371/journal.pone.0188502)
Supplement: S1 Text — (DOCX) [file pone.0188502.s001.docx]

**Supplementary list 1 – Case control questionnaire**

1. Date of filling out the questionnaire

**General**

1. What is your sex?
2. What is your birthdate?
3. What is your postal code? (4 out of 6 characters)
   1. What is your country of birth?
   2. If you were born outside the Netherlands, were you adopted?
4. In which country were your parents born?
   1. Did you move the last two years?
   2. If yes, what was your previous postal code? (4 out of 6 characters)
5. What is your sexual preference?
6. In your household, are you the one who regularly cooks?
7. Do you wash your hands when preparing the food?
   1. Are you vegetarian?
   2. If no, do you eat raw meat?
8. Do you visit one of the following locations? (Swimming pool, beach/sea, river/lake, sauna, whirlpool, pedicure, beauty salon)
9. Did you have a tattoo or piercing in the last twelve months?

**Household**

1. How many people are in your household?
2. Which of the following objects do you regularly share among your household members? (towels, wash cloths, bedsheets, shaving razors, toothbrushes, combs, creams, other, none)
3. Was someone in your household hospitalized the last year?

**MRSA**

- 1. Have you ever been MRSA positive?
  2. In the last twelve months?
  3. Do you know the exact period?
  4. Who detected the MRSA? (Hospital, long term care facility, general practitioner, other)
  5. Have you ever been treated against MRSA with an ‘eradication therapy’?
  6. Did this get rid of the MRSA?
  7. During eradication therapy, were your household members screened?
  8. Were they also treated?

**General health**

- 1. Do you sport?
  2. What type of sport? (team, individual, contact, with animals)

1. Do you smoke?
   1. Do you dialyze?
   2. If so, have you been dialyzed abroad?
   3. Do you know if you have/had contact with a MRSA carrier?
   4. If so, where did you have contact with this MRSA carrier?
   5. If elsewhere, where exactly?
   6. What was your relation compared to this MRSA carrier?
   7. Do you have close contact with certain people? (inside the household, outside or with people from inside and outside the household)
   8. Have you ever been contacted in regards to contact tracing for another MRSA carrier?
   9. Were you then found to be MRSA positive?
   10. Did you use antibiotics in the last twelve months?
   11. If so, how often and when?
   12. If so, do you remember the names?
   13. Do you have a skin disease diagnosed by a doctor?
   14. If so, is this eczema?
   15. Do you have a chronic disease?
   16. If so, to which group does your chronic disease belong? (lungs, cardiac/heart, auto-immune, other metabolic, diabetes, chronic infection, congenital, other, don’t know)
   17. Do you happen to know the name of your chronic disease?
   18. Do you use immunosuppressive medications?
   19. If so, do you remember the names of these medications?
   20. Did you receive ambulant care the last two years?
   21. If so, how often?
   22. If so, which kind? (homecare, general practitioner, mental healthcare, obstetric care, maternity, supervised living, physiotherapy, dispensary, other)
2. Did you visit an outpatient department of a Dutch hospital in the last twelve months?
3. Were you admitted to a Dutch hospital or nursing home in the last twelve months?
4. Were you care for on a department in a Dutch hospital or nursing home in the last twelve months where there was a known MRSA outbreak?
5. Did you come into contact with a MRSA carrier in a Dutch hospital or nursing home?

**Work**

1. Which situation applies to you? (Employed, retired, standing entrepreneur, student, jobless, going to school, volunteer job, <4 years old)
2. In which sector do/did you work? (Healthcare, animals, shipping, other)
3. In which sector does/did your partner work? (healthcare, animals, shipping, other)
4. In Which sector does/did your household members work? (healthcare, animals, shipping, other)

Following questions only for those working in healthcare

- 1. Have you come into contact with a MRSA carrier?
  2. Did you take infection prevention precautions?
  3. Was MRSA detected at you after you had contact with a MRSA carrier?

1. Did you accompany patients in the last twelve months when they were transferred from a hospital abroad to one in the Netherlands? (Or the other way around)

**Animal Contact**

- 1. Did you have contact with pigs or veal calves outside your work?
  2. If yes, with pigs, veal calves or both?
  3. Where did you come into contact with these animals?
  4. Did you partner or one of your household members come into contact with pigs or veal calves outside work?
  5. If yes, where did they come into contact with these animals?
  6. Do you keep pets or farm animals?
  7. If so, which ones?

1. Do you ride or take care of horses?
2. Do you visit farms or petting zoos?

**Abroad**

- 1. Have you been abroad the previous year?
  2. If so, please name the countries you travelled to in your last five trips abroad within a maximum timespan of two years. And whether or not you visited a hospital then.
  3. If you visit a hospital abroad in the last two years, what was the reason? (Visit a patient, small surgery, outpatient clinic visit, admitted to hospital as patient, for work, other)
  4. Did you become MRSA positive after your visit to an abroad hospital in the last two years?
  5. Did you visit a Dutch hospital after your abroad hospital visit?
  6. Have you *ever* been in an abroad hospital?
  7. Did a household or family member become MRSA positive after an abroad hospital visit? (in the last two years)
  8. Has a household or family member been to a Dutch hospital after visit an abroad hospital? (in the last two years)
  9. Has a household or family member *ever* been to an abroad hospital?
