# Supplementary material for: Newly identified risk factors for MRSA carriage in The Netherlands
Source: PLoS One. 2017 Nov 30;12(11):e0188502. doi: 10.1371/journal.pone.0188502 (PMC5708665; doi:10.1371/journal.pone.0188502)

Logistic Regression Results

The LOGISTIC Procedure

Model Information		
Data Set	WORK.SORTTEMPTABLESORTED	
Response Variable	Rx	Rx
Number of Response Levels	2	
Model	binary logit	
Optimization Technique	Fisher's scoring	

Number of Observations Read	899
Number of Observations Used	834

Response Profile		
Ordered Value	Rx	Total Frequency
1	Control	627
2	MUO	207

Probability modeled is Rx='MUO'.

Note: 65 observations were deleted due to missing values for the response or explanatory variables.

Backward Elimination Procedure

Step 0. The following effects were entered:

Intercept N45 N214 N123 C11A C80A N136 N81

Model Convergence Status
Convergence criterion (GCONV=1E-8) satisfied.

Model Fit Statistics		
Criterion	Intercept Only	Intercept and Covariates
AIC	936.665	762.309
SC	941.391	800.119
-2 Log L	934.665	746.309

R-Square	0.2022	Max-rescaled R-Square	0.3000

Testing Global Null Hypothesis: BETA=0			
Test	Chi-Square	DF	Pr > ChiSq
Likelihood Ratio	188.3555	7	<.0001
Score	191.2730	7	<.0001
Wald	150.2060	7	<.0001

Logistic Regression Results

The LOGISTIC Procedure

Model Convergence Status
Convergence criterion (GCONV=1E-8) satisfied.

Model Fit Statistics		
Criterion	Intercept Only	Intercept and Covariates
AIC	936.665	760.571
SC	941.391	793.655
-2 Log L	934.665	746.571

R-Square	0.2019	Max-rescaled R-Square	0.2996

Testing Global Null Hypothesis: BETA=0			
Test	Chi-Square	DF	Pr > ChiSq
Likelihood Ratio	188.0934	6	<.0001
Score	190.9509	6	<.0001
Wald	149.9288	6	<.0001

Residual Chi-Square Test		
Chi-Square	DF	Pr > ChiSq
0.2636	1	0.6076

Step 2. Effect N214 is removed:

Model Convergence Status
Convergence criterion (GCONV=1E-8) satisfied.

Model Fit Statistics		
Criterion	Intercept Only	Intercept and Covariates
AIC	936.665	759.727
SC	941.391	788.084
-2 Log L	934.665	747.727

R-Square	0.2008	Max-rescaled R-Square	0.2979

Testing Global Null Hypothesis: BETA=0			
Test	Chi-Square	DF	Pr > ChiSq
Likelihood Ratio	186.9382	5	<.0001
Score	190.0236	5	<.0001
Wald	149.1911	5	<.0001

Residual Chi-Square Test		
Chi-Square	DF	Pr > ChiSq
1.4777	2	0.4777

Step 3. Effect N123 is removed:

Logistic Regression Results

The LOGISTIC Procedure

Model Convergence Status
Convergence criterion (GCONV=1E-8) satisfied.

Model Fit Statistics		
Criterion	Intercept Only	Intercept and Covariates
AIC	936.665	760.012
SC	941.391	783.643
-2 Log L	934.665	750.012

R-Square	0.1986	Max-rescaled R-Square	0.2947

Testing Global Null Hypothesis: BETA=0			
Test	Chi-Square	DF	Pr > ChiSq
Likelihood Ratio	184.6528	4	<.0001
Score	187.8429	4	<.0001
Wald	148.4386	4	<.0001

Residual Chi-Square Test		
Chi-Square	DF	Pr > ChiSq
3.8295	3	0.2805

Note: No (additional) effects met the 0.05 significance level for removal from the model.

Summary of Backward Elimination						
Step	Effect Removed	DF	Number In	Wald Chi-Square	Pr > ChiSq	Variable Label
1	N45	1	6	0.2635	0.6077	
2	N214	1	5	1.1952	0.2743	
3	N123	1	4	2.3185	0.1278	

Analysis of Maximum Likelihood Estimates					
Parameter	DF	Estimate	Standard Error	Wald Chi-Square	Pr > ChiSq
Intercept	1	-2.3473	0.1527	236.3330	<.0001
C11A	1	0.8589	0.2519	11.6264	0.0007
C80A	1	1.4592	0.3637	16.1001	<.0001
N136	1	0.8165	0.2459	11.0299	0.0009
N81	1	2.0972	0.1861	126.9666	<.0001

Odds Ratio Estimates			
Effect	Point Estimate	95% Wald Confidence Limits	
C11A	2.361	1.441	3.867
C80A	4.303	2.109	8.776
N136	2.263	1.397	3.664
N81	8.143	5.654	11.728

Logistic Regression Results

The LOGISTIC Procedure

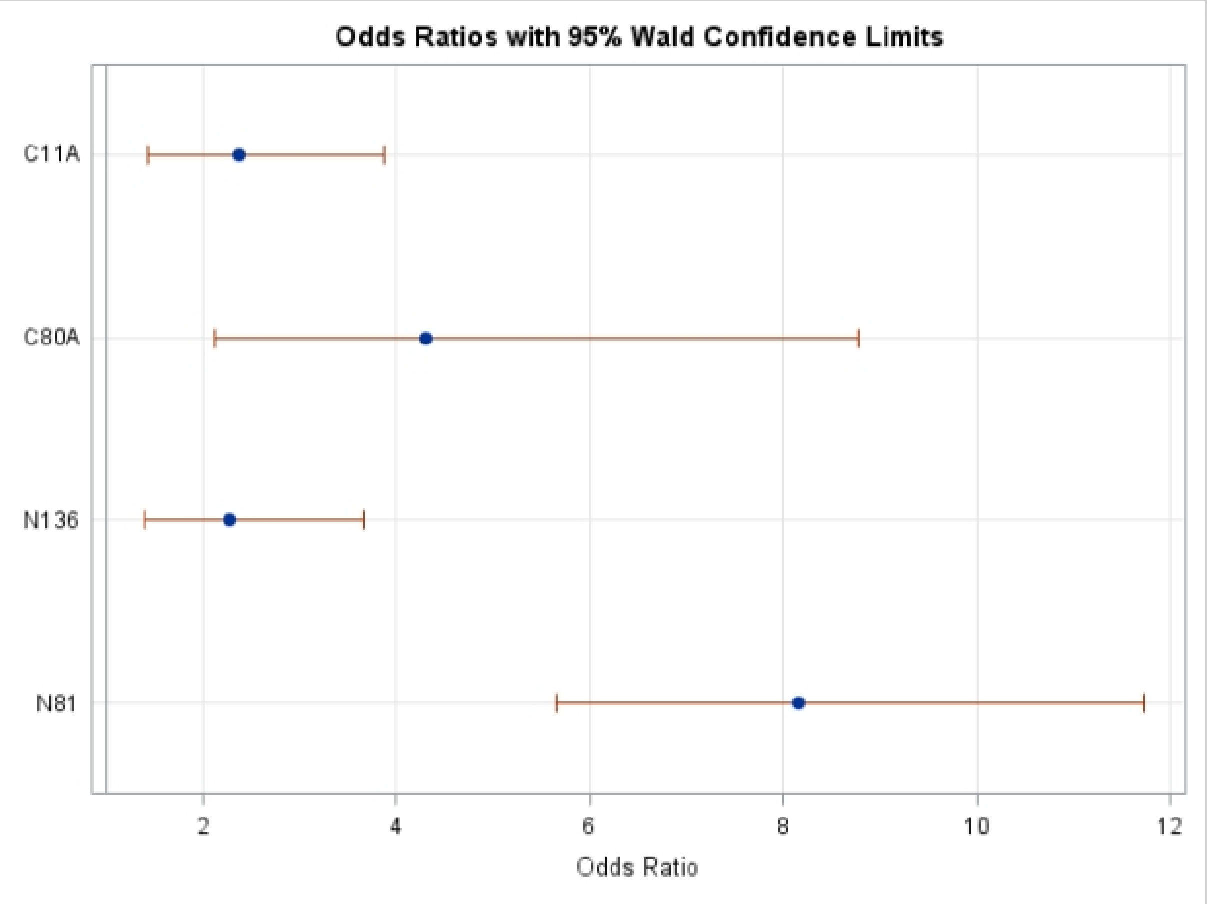

Association of Predicted Probabilities and Observed Responses			
**Percent Concordant**	69.7	**Somers' D**	0.573
**Percent Discordant**	12.4	**Gamma**	0.698
**Percent Tied**	17.9	**Tau-a**	0.214
**Pairs**	129789	**c**	0.786

Logistic Regression Results

The LOGISTIC Procedure

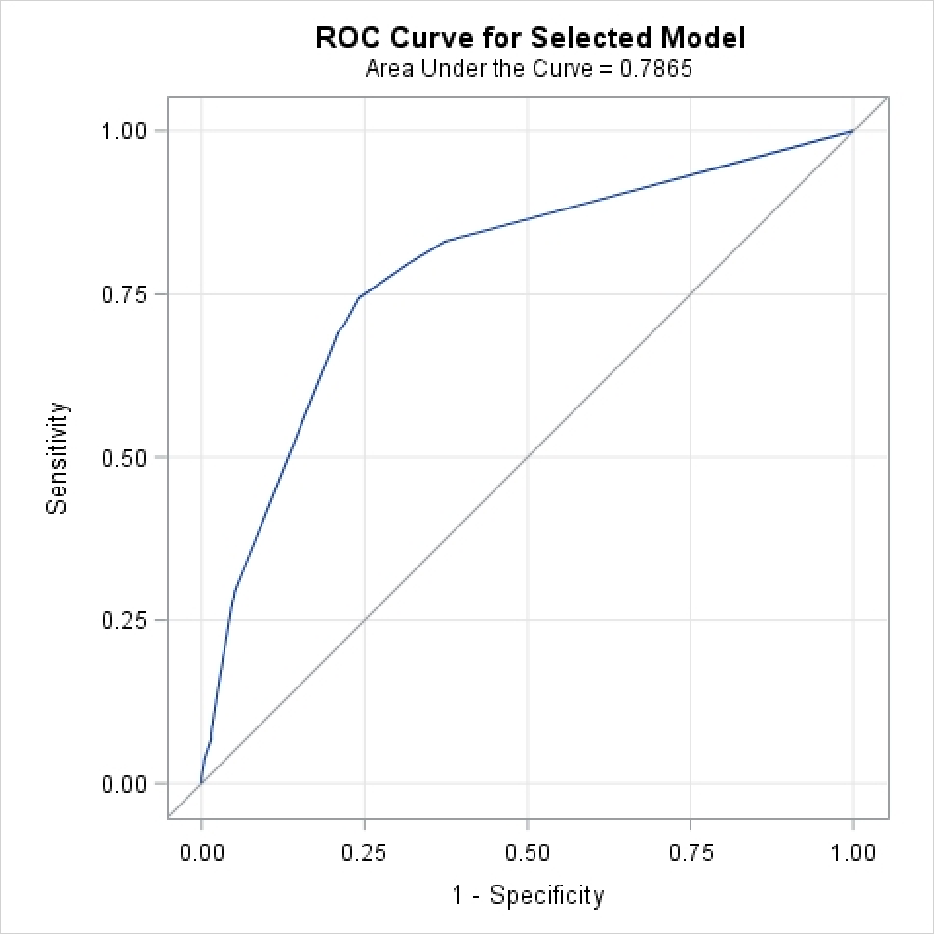

Logistic Regression Results

The LOGISTIC Procedure

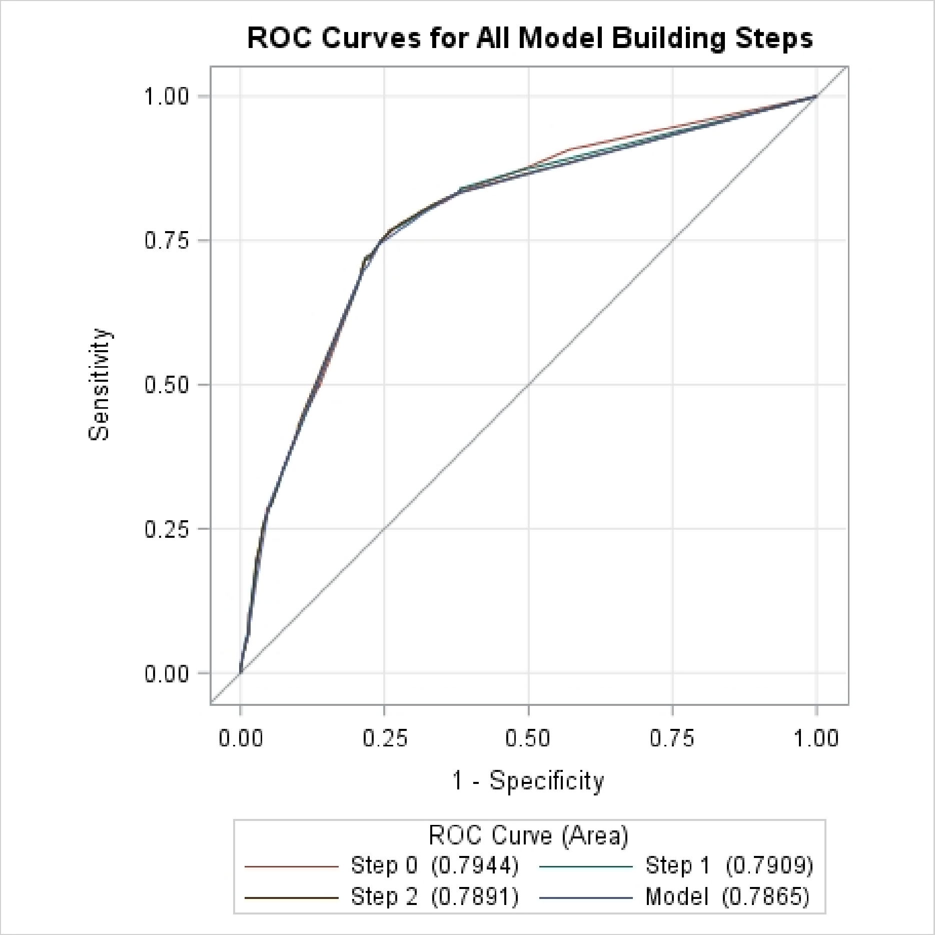

Partition for the Hosmer and Lemeshow Test					
		Rx = MUO		Rx = Control	
Group	Total	Observed	Expected	Observed	Expected
1	428	35	37.36	393	390.64
2	101	18	18.34	83	82.66
3	31	11	9.50	20	21.50
4	181	82	79.24	99	101.76
5	93	61	62.57	32	30.43

Hosmer and Lemeshow Goodness-of-Fit Test		
Chi-Square	DF	Pr > ChiSq
0.8016	3	0.8491

Logistic Regression Results

The LOGISTIC Procedure

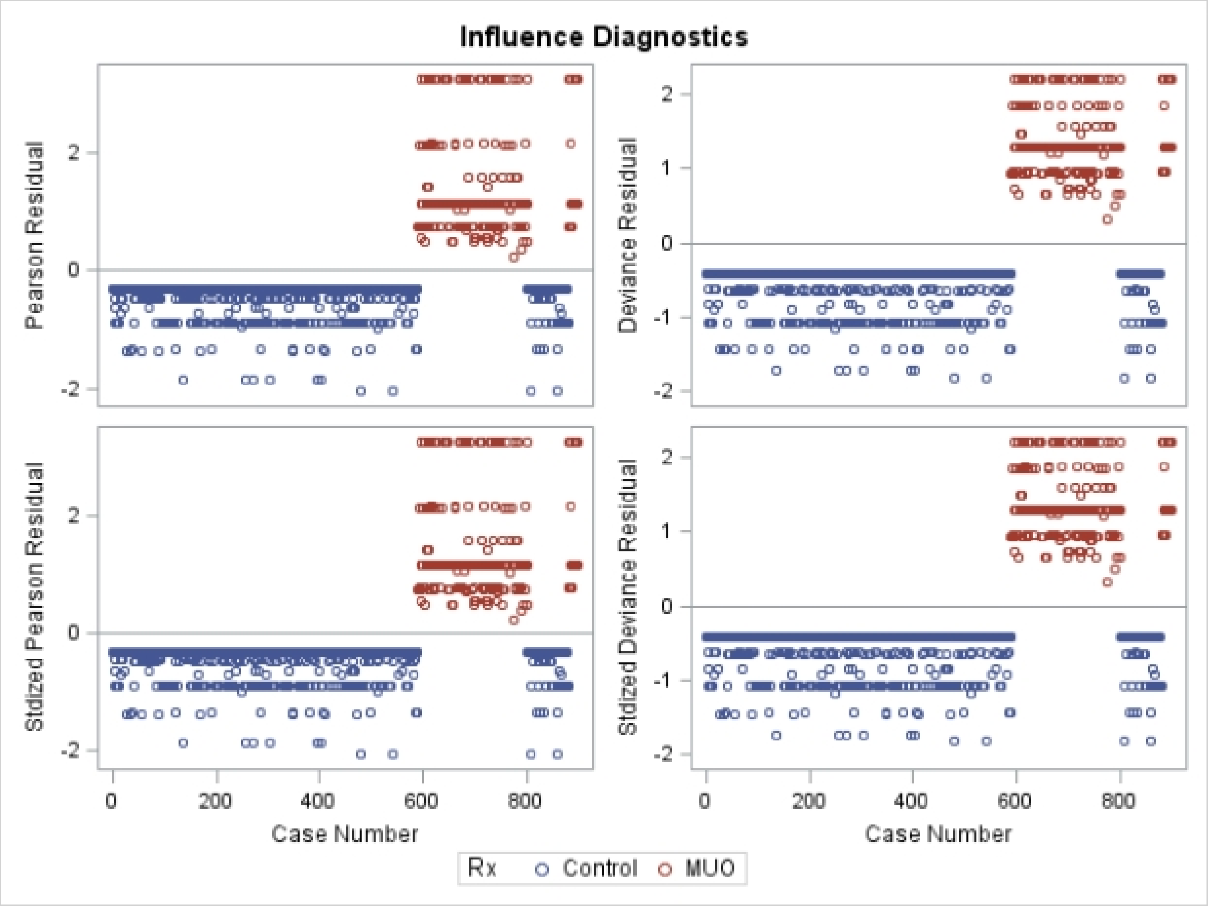

Logistic Regression Results

The LOGISTIC Procedure

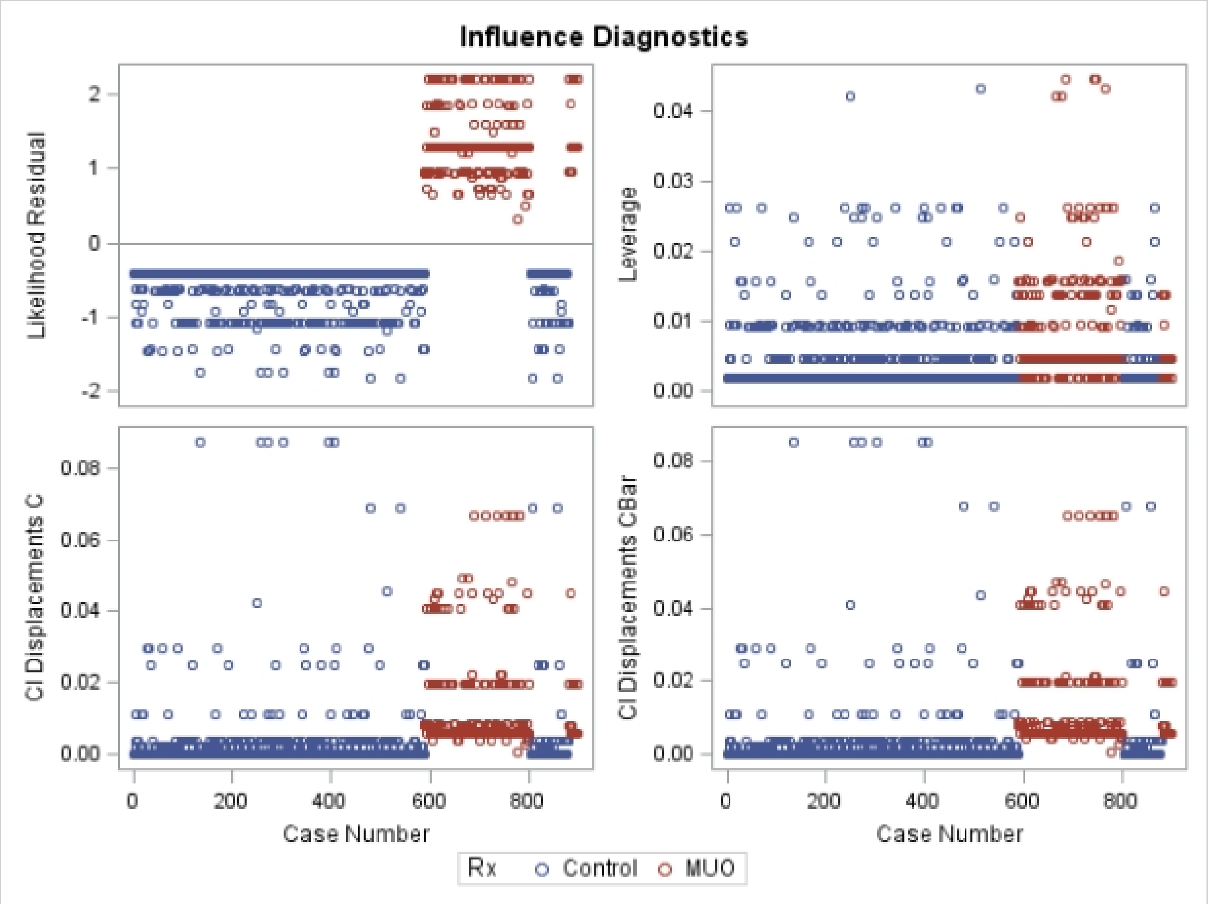

Logistic Regression Results

The LOGISTIC Procedure

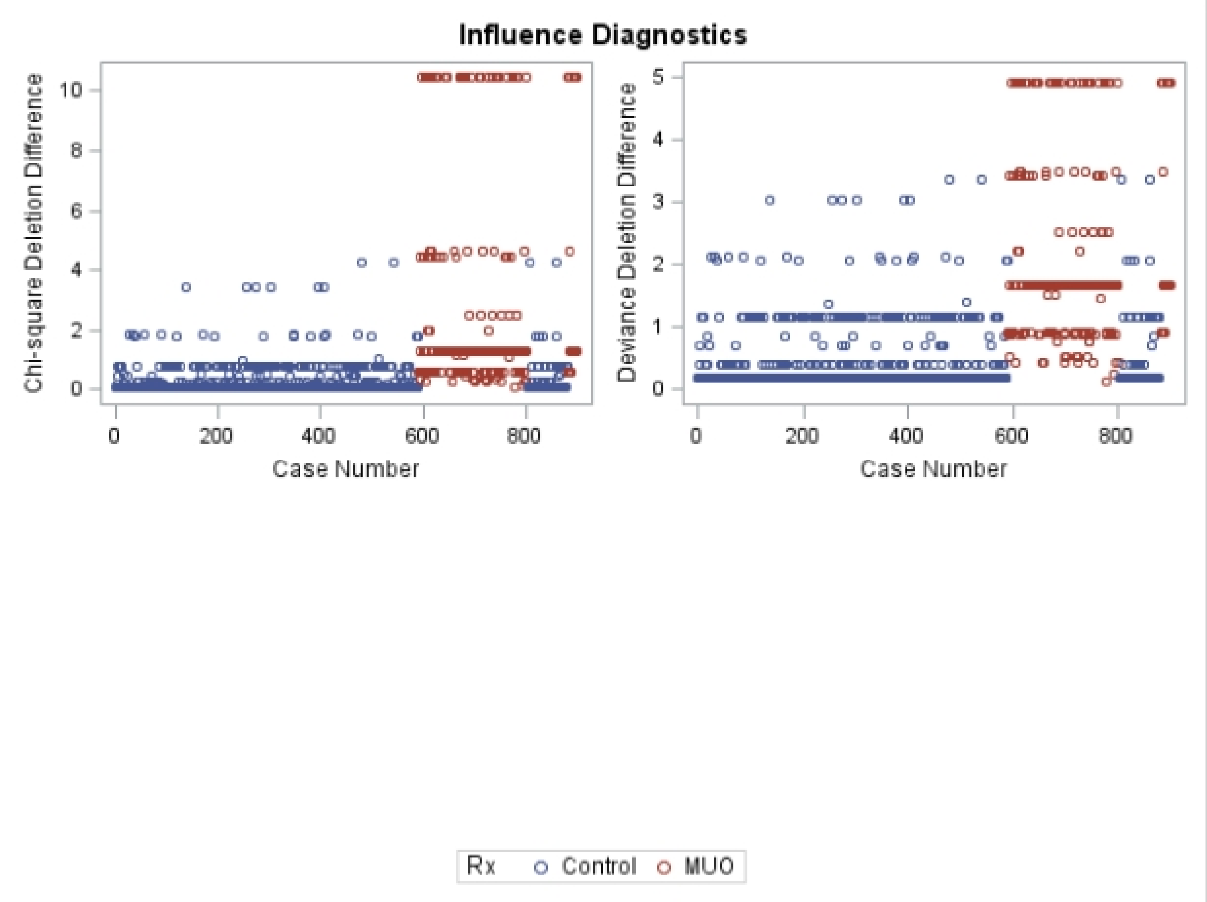

Logistic Regression Results

The LOGISTIC Procedure

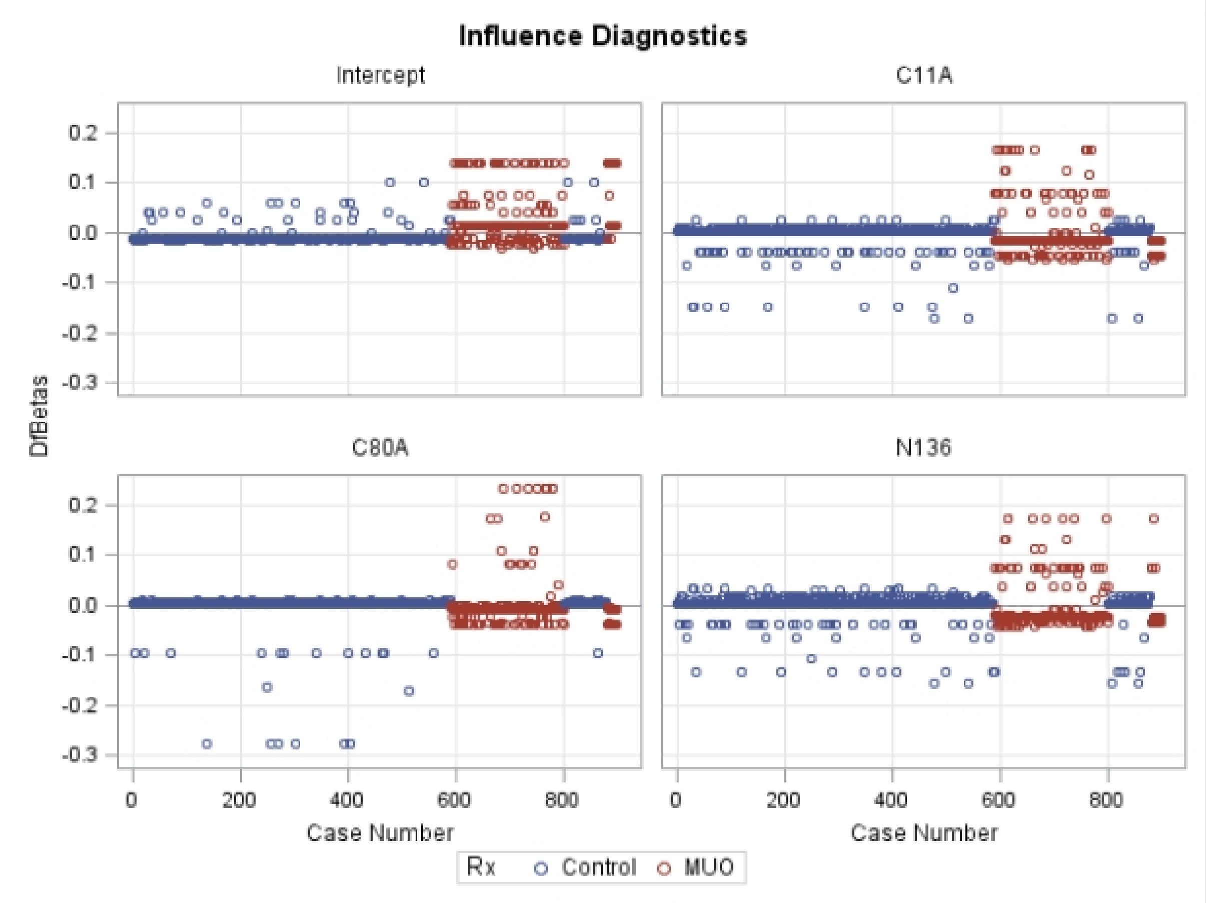

Logistic Regression Results

The LOGISTIC Procedure

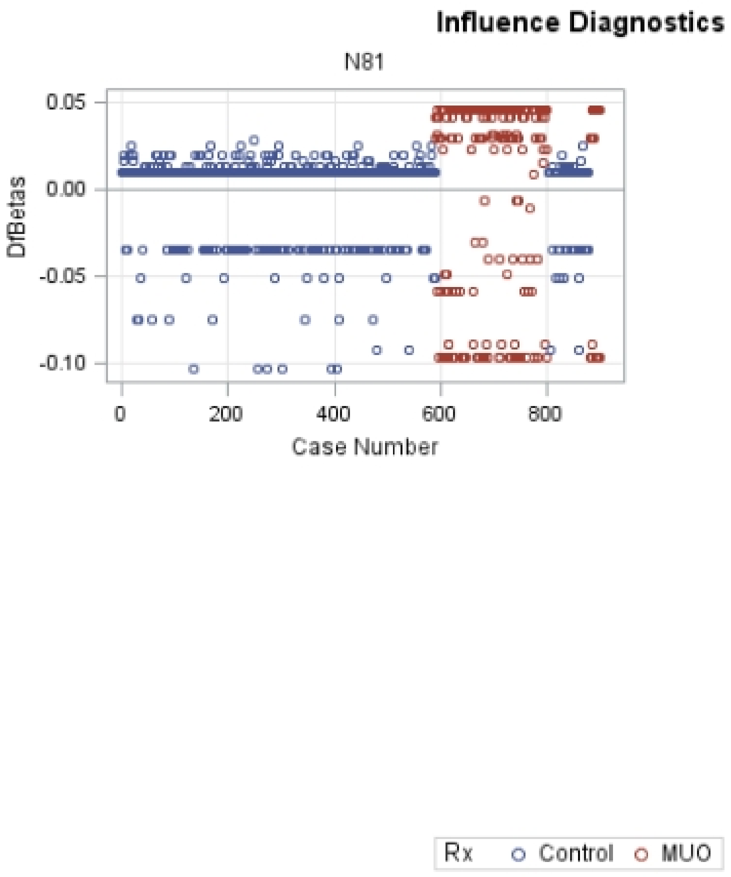

Logistic Regression Results

The LOGISTIC Procedure

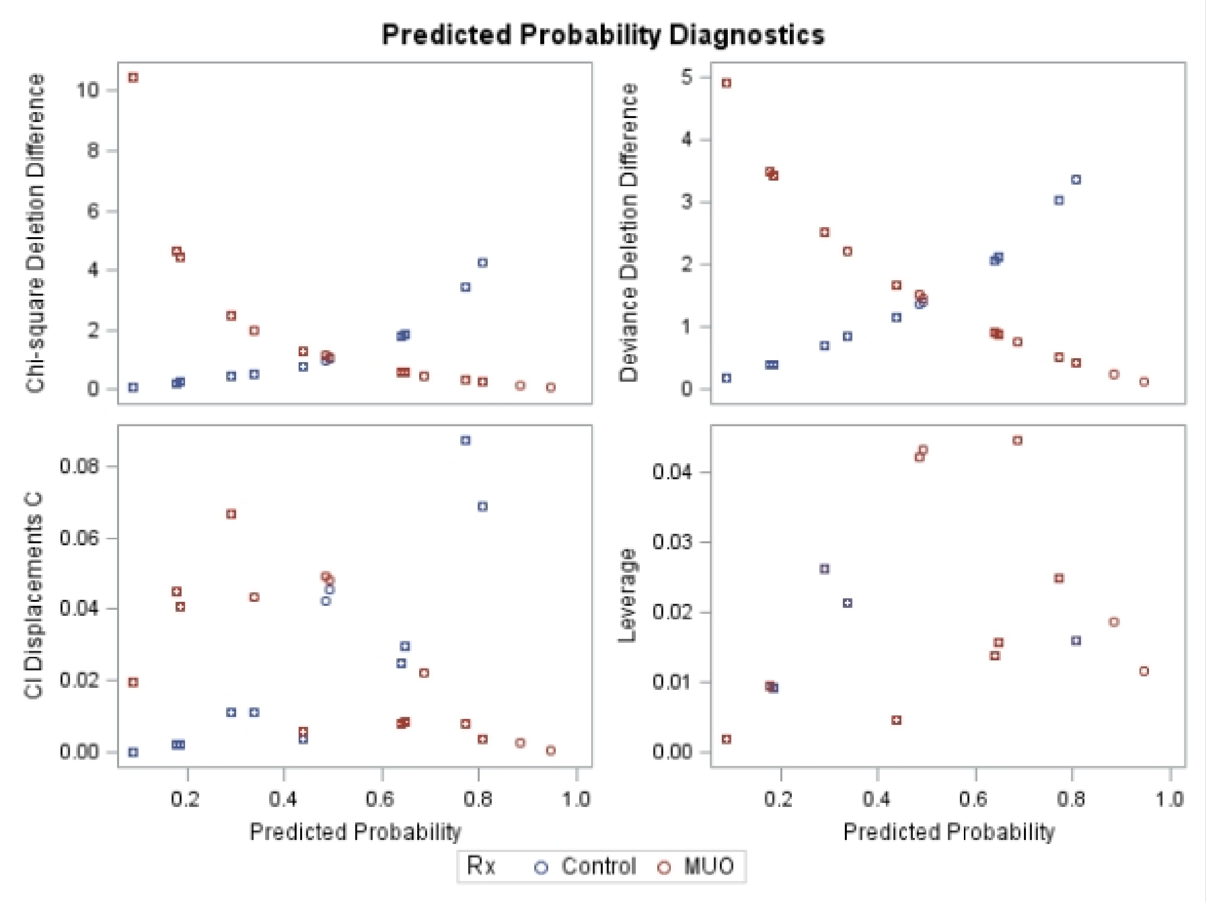

Logistic Regression Results

The LOGISTIC Procedure

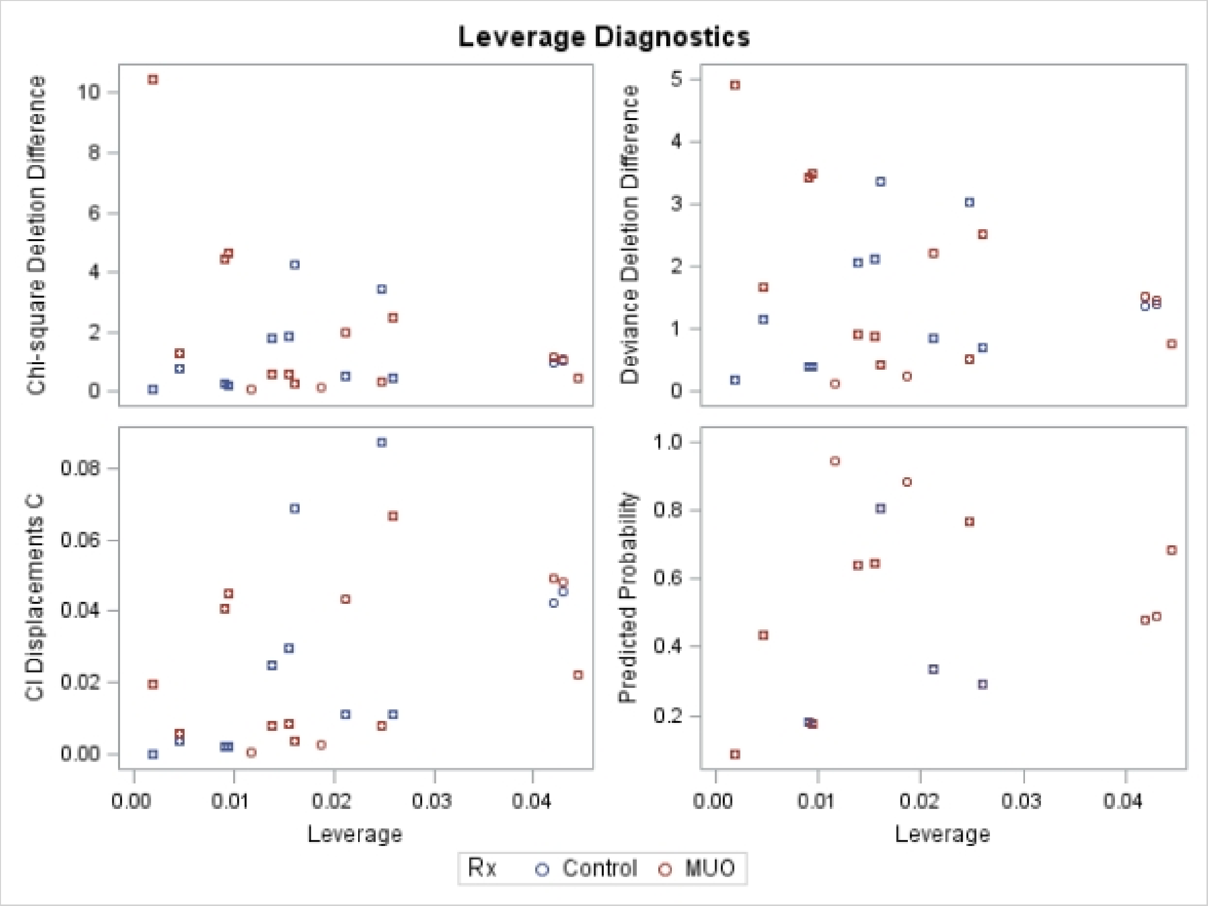

Logistic Regression Results

The LOGISTIC Procedure

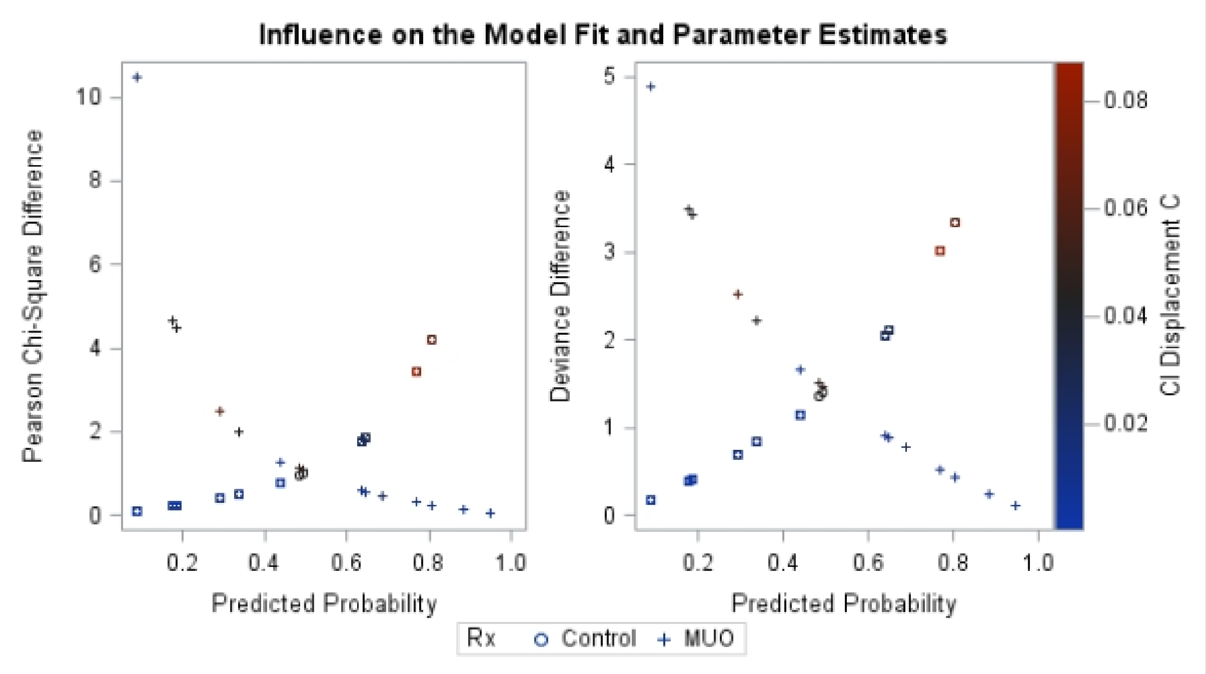

Logistic Regression Results

The LOGISTIC Procedure

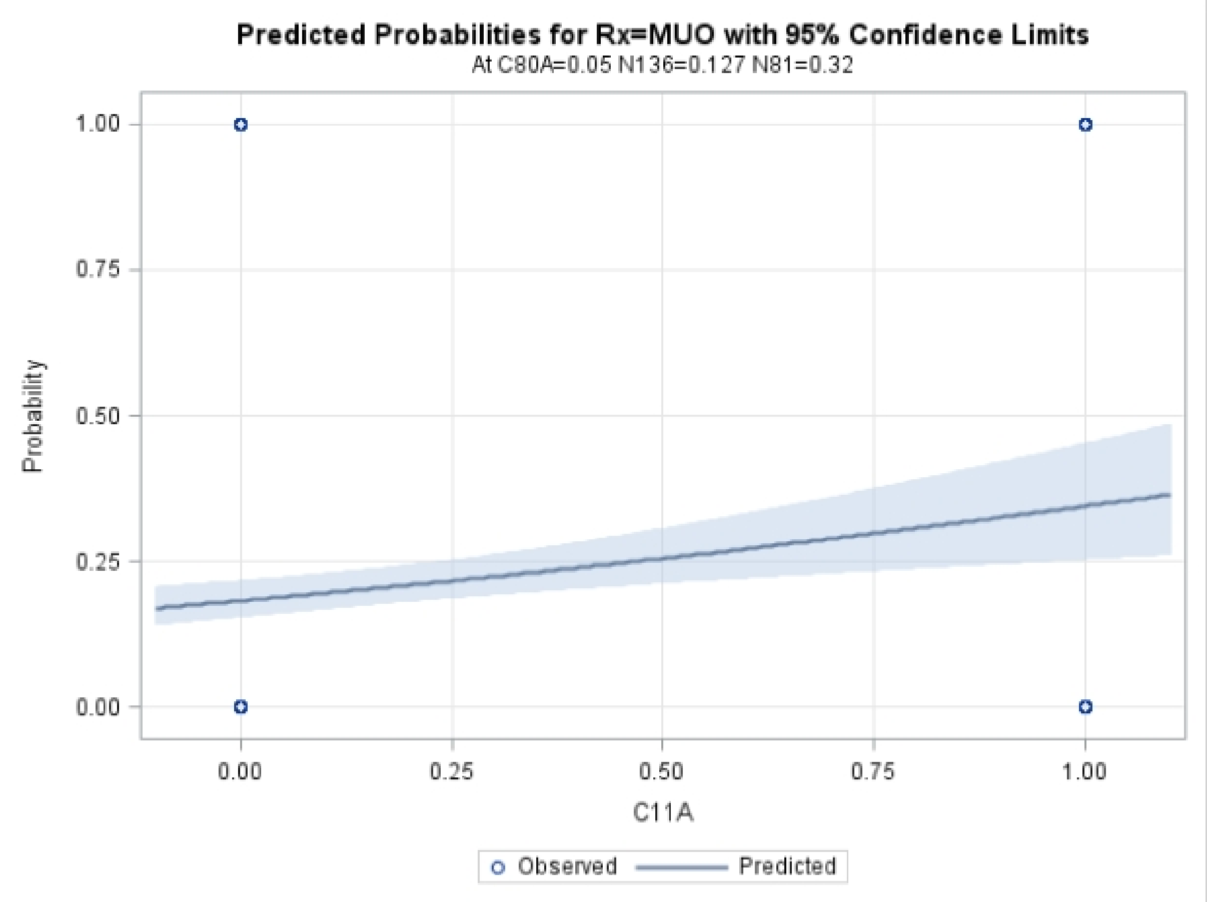

Supplement: S3 Text — (PDF) [file pone.0188502.s003.pdf]
